# Durable response to olaparib plus continued PD-1 blockade in germline *PALB2*-mutated triple-negative breast cancer with leptomeningeal metastasis: A case report

**DOI:** 10.1097/MD.0000000000050334

**Published:** 2026-08-21

**Authors:** Yulei Wang, Jiali Lin, Yi Zhang, Bo Liang, Xin Lin, Xiangqing Chen, Lijuan Guo, Yifang Zhang, Yuxia Chen, Guochun Zhang

**Affiliations:** a Department of Breast Surgery, Guangdong Provincial People’s Hospital, Guangdong Academy of Medical Sciences, Southern Medical University, Guangzhou, Guangdong, China; b Department of Breast Cancer, Guangdong Provincial People’s Hospital’s Nanhai Hospital, Foshan, Guangdong, China; c Department of Breast Surgery, Maoming People’s Hospital, Maoming, Guangdong, China; d Department of Radiology, Maoming People’s Hospital, Maoming, Guangdong, China.

**Keywords:** case report, leptomeningeal metastasis, olaparib, *PALB2* mutation, tislelizumab, triple-negative breast cancer

## Abstract

**Rationale:**

Triple-negative breast cancer (TNBC) with germline *PALB2 (gPALB2*) mutations represents a rare but actionable subset sensitive to poly(ADP-ribose) polymerase (PARP) inhibition. However, clinical evidence supporting PARP inhibition combined with programmed cell death protein 1 (PD-1) blockade after progression on prior immunotherapy remains limited, particularly in central nervous system-involved disease.

**Patient concerns:**

A 61-year-old woman initially diagnosed with stage I TNBC developed chest wall recurrence and occipital bone metastasis after a 2-year disease-free interval. The disease subsequently progressed to spinal leptomeningeal metastasis, causing complete bilateral lower-limb paralysis.

**Diagnoses:**

Recurrent metastatic TNBC with programmed death-ligand 1 expression and leptomeningeal and multiple osseous metastases was diagnosed. Pharmacogenomic profiling identified a pathogenic *gPALB2* p.L484* mutation.

**Interventions:**

First-line liposomal doxorubicin achieved 9 months of disease control. Second-line nab-paclitaxel plus tislelizumab, a PD-1 inhibitor, provided a progression-free survival of only 2 months. Third-line carboplatin plus tislelizumab induced rapid neurological improvement, but carboplatin was discontinued after 2 months due to hematologic toxicity. Maintenance tislelizumab monotherapy was continued for an additional 5 months until neurological deterioration and a new femoral metastasis indicated disease progression. Olaparib was subsequently added to continued tislelizumab.

**Outcomes:**

Following olaparib initiation, bilateral lower-limb muscle strength improved from Medical Research Council grade 0 to grade 2. Follow-up imaging demonstrated marked regression of the leptomeningeal and osseous lesions. The patient achieved a durable progression-free survival of approximately 40 months with acceptable tolerability.

**Lessons:**

Identification of a *gPALB2* mutation informed the addition of olaparib to continued tislelizumab, leading to sustained disease control in this patient. Although the relative contributions of each component cannot be determined from a single case, this hypothesis-generating observation suggests that PARP inhibitor–based strategies and continuation of PD-1 blockade beyond progression warrant further investigation in carefully selected patients with *gPALB2*-mutated TNBC with central nervous system involvement.

## 1. Introduction

Triple-negative breast cancer (TNBC) accounts for approximately 15% of breast cancers and is defined by the absence of estrogen receptor (ER), progesterone receptor (PR), and human epidermal growth factor receptor 2 (HER2) expression.^[1]^ Its aggressive clinical course, high risk of early relapse, and limited targeted options result in a poor prognosis.^[2]^ The advent of precision oncology and pharmacogenomics has enabled genetic profiling to inform individualized treatment decisions, offering new opportunities for targeted therapy in selected patients. Immune checkpoint inhibitors (ICIs) and poly(ADP-ribose) polymerase (PARP) inhibitors are promising strategies for homologous recombination repair (HRR)–deficient tumors.^[3,4]^ PARP inhibitors, such as olaparib, are now approved as monotherapy for germline *BRCA1/2*-mutated, HER2-negative breast cancer.^[5,6]^ Partner and localizer of BRCA2 (*PALB2*), a key component of the HRR pathway, interacts with *BRCA2* and plays an essential role in maintaining genomic stability.^[7,8]^ Germline mutations in *PALB2 (gPALB2*) may be associated with unfavorable long-term outcomes^[9]^ and confer a pharmacogenomic vulnerability to PARP inhibition through synthetic lethality.^[10]^ While single-agent clinical activity of PARP inhibitors in *gPALB2*-mutated breast cancer has been demonstrated,^[11,12]^ evidence regarding combinations with programmed cell death protein 1 (PD-1) or programmed death-ligand 1 (PD-L1) blockade remains notably limited. Here, we report a patient with *gPALB2*-mutated TNBC and leptomeningeal metastasis (LM) who achieved an unusually prolonged progression-free survival (PFS) of approximately 40 months following the addition of olaparib to continued tislelizumab therapy upon progression on prior anti-PD-1 maintenance monotherapy.

## 2. Case presentation

**Figure 1.**
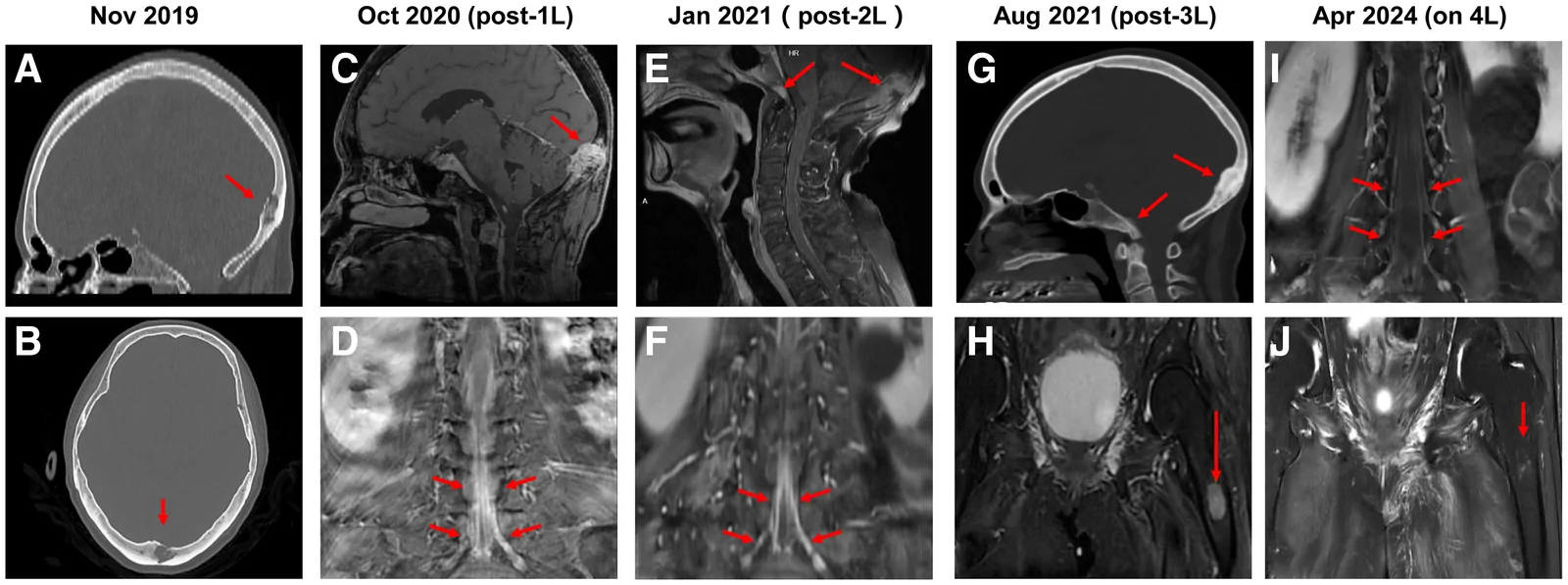
Key radiological findings during the clinical course. (A, B) Sagittal (A) and axial (B) head CT (bone window) in November 2019 showing the initially detected occipital bone metastasis. (C, D) Sagittal cranial MRI in October 2020 confirming the occipital lesion (C) and coronal lumbosacral enhanced MRI revealing a new leptomeningeal metastasis involving the cauda equina (D). (E, F) Sagittal cervical enhanced MRI in January 2021 showing a new clival lesion with the occipital metastasis (E) and coronal lumbosacral enhanced MRI demonstrating progression of leptomeningeal disease (F). (G, H) Sagittal head CT (bone window) in August 2021 showing osteoblastic changes with partial relief of occipital/clival lesions (G) and coronal sacroiliac MRI showing a new proximal left femoral metastasis (2.3 × 1.3 cm) (H). (I, J) Coronal spinal and femoral enhanced MRI in April 2024 showing sustained regression of leptomeningeal metastasis (I) and resolution of the femoral lesion (J). Red arrows indicate the metastatic lesions. CT = computed tomography, MRI = magnetic resonance imaging.

A 61-year-old woman underwent modified radical mastectomy in 2017 for pT1cN0M0 invasive ductal carcinoma (ER−/PR−/HER2−). She received 6 cycles of adjuvant liposomal paclitaxel plus cyclophosphamide. After a 24-month disease-free interval, she developed occipital bone metastasis (Fig. 1A and B) and chest wall recurrence in 2019. The chest wall lesion was resected and irradiated at another hospital, and histopathology reconfirmed TNBC.

She then received first-line liposomal doxorubicin with ibandronate, achieving a PFS of 9 months. Upon progression, she developed paraplegia and, in October 2020, was referred to our hospital for further evaluation. Thereafter, incadronate, a third-generation bisphosphonate, was maintained throughout all subsequent lines of therapy for metastatic disease with bone involvement. Magnetic resonance imaging confirmed the known occipital bone metastasis (Fig. 1C) and revealed new LM predominantly involving the spinal meninges surrounding the cauda equina (Fig. 1D), accounting for complete bilateral lower-limb paralysis (Medical Research Council [MRC] grade 0).

**Figure 2.**
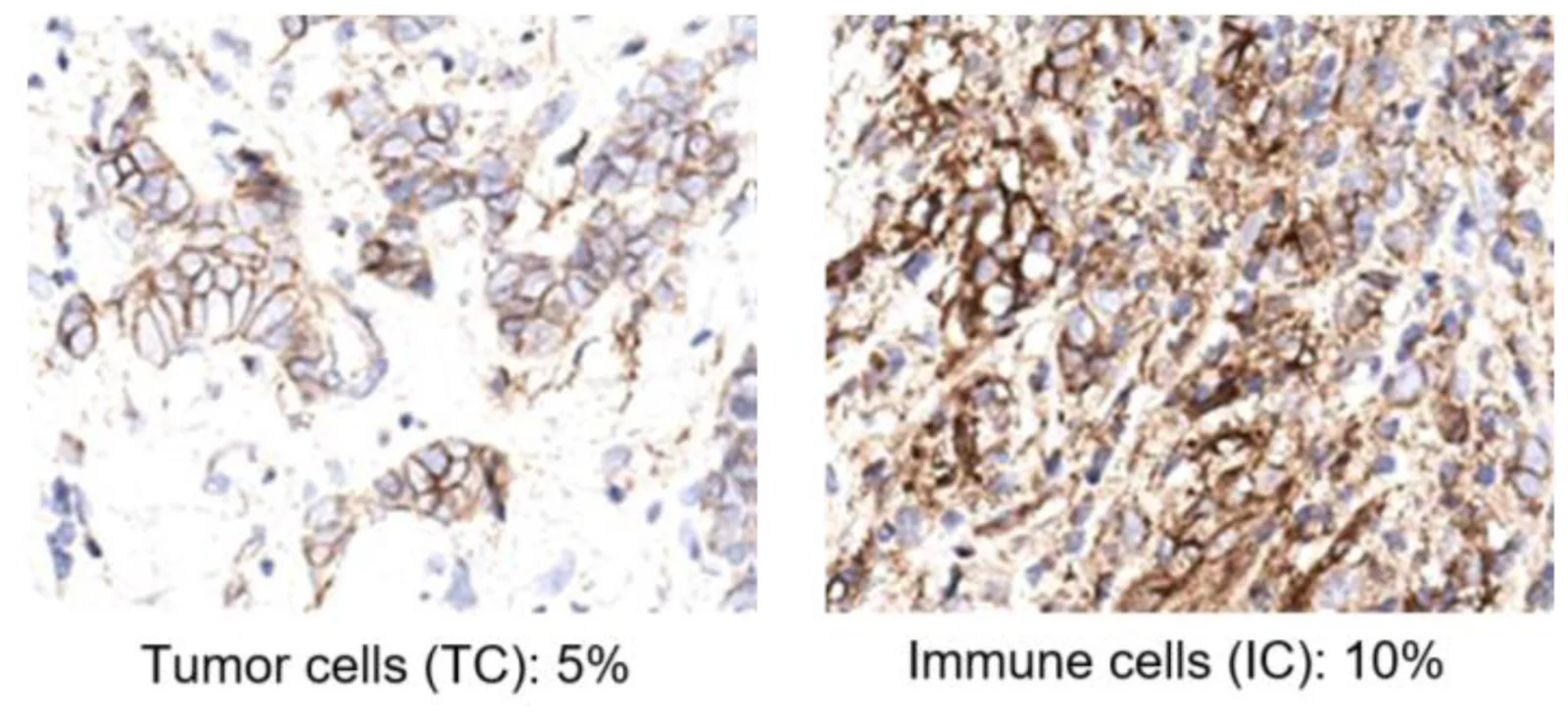
PD-L1 immunohistochemical staining. Tumor tissue from the recurrent right chest wall lesion stained with the SP142 antibody, showing 5% positivity in tumor cells and 10% positivity in tumor-infiltrating immune cells. PD-L1 = programmed death-ligand 1.

Pharmacogenomic profiling was performed using paired deoxyribonucleic acid from the recurrent chest wall lesion and peripheral blood with a 520-gene panel that included 62 hereditary cancer susceptibility genes for germline variant analysis.^[13,14]^ This analysis identified a heterozygous nonsense mutation in *PALB2* (exon 4, c.1451T>G, p.L484*), representing the only pathogenic germline variant detected. Additional somatic alterations included *TP63* (p.V240L), *CSF1R* (p.P570fs), and amplification of *MDC1* and *BLM*. The tumor was microsatellite-stable with a low tumor mutational burden (2.99 mut/Mb). PD-L1 immunohistochemistry (SP142) revealed 5% positivity in tumor cells and 10% in tumor-infiltrating immune cells (Fig. 2), supporting eligibility for ICI therapy.^[15,16]^

Second-line nab-paclitaxel plus tislelizumab, an anti-PD-1 monoclonal antibody,^[17]^ provided only transient neurologic improvement before bilateral lower-limb muscle strength declined to MRC grade 0 (complete paralysis). Progression was documented with a new clival lesion (Fig. 1E) and mild leptomeningeal progression (Fig. 1F), with a PFS of 2 months.

Third-line therapy with carboplatin plus tislelizumab was commenced, but carboplatin was discontinued after 2 months due to severe hematologic toxicity, and maintenance tislelizumab monotherapy was continued for an additional 5 months. Remarkably, within 2 weeks of third-line treatment initiation, her bilateral lower-limb muscle strength improved from MRC grade 0 to grade 2, with this improvement sustained for approximately 7 months before subsequently deteriorating to grade 0. The abrupt neurological decline prompted evaluation; however, due to refusal of contrast-enhanced imaging, only plain magnetic resonance imaging and computed tomography scans were obtained. Although leptomeningeal progression could not be confirmed, imaging showed partial relief of the clival and occipital bone metastases (Fig. 1G) but revealed a new metastatic lesion in the proximal left femur (Fig. 1H). Together with the neurological deterioration, this finding indicated overall disease progression, with a PFS of 7 months.

Fourth-line therapy with olaparib (300 mg bid) plus tislelizumab (200 mg q3w) was initiated in September 2021, after the patient declined further chemotherapy. This decision was directly guided by the detection of the *gPALB2* mutation (p.L484*), using the pharmacogenomic data and supported by evidence from the TBCRC 048 trial.^[11]^ Within 1 month, bilateral lower-limb strength improved from MRC grade 0 to grade 2 and remained stable without new symptoms or hematotoxicity. Given this sustained clinical benefit, interim imaging assessments were deferred. A follow-up evaluation was eventually performed in April 2024 and revealed osteoblastic changes in the clival and occipital bone lesions. The evaluation also showed marked regression of LM surrounding the cauda equina (Fig. 1I) and dramatic resolution of the previously identified left femoral lesion (Fig. 1J), with no new visceral metastasis detected. The regimen was continued until November 2024. In January 2025, the patient died from infection-related septic shock secondary to pressure ulcer complications. The PFS achieved with this low-toxicity regimen was 40 months.

## 3. Discussion

This case illustrates the potential clinical value of pharmacogenomic profiling in guiding treatment selection for advanced TNBC. Identification of a pathogenic *gPALB2* mutation informed the addition of olaparib to continued tislelizumab, which was followed by prolonged disease control and neurological improvement after limited benefit from prior PD-1–based regimens. The patient’s treatment course and clinical timeline are summarized in Table 1. Although PD-L1 positivity supported the use of ICI therapy,^[15,16]^ the patient derived only transient benefit from her initial PD-1–based regimen of nab-paclitaxel plus tislelizumab, with a PFS of 2 months. However, prompt neurological recovery was observed after switching the chemotherapy backbone to carboplatin. Although carboplatin was discontinued after 2 months because of hematologic toxicity, the neurological improvement was sustained during an additional 5 months of tislelizumab monotherapy, until neurological deterioration and a new femoral metastasis indicated overall disease progression. This clinical course is consistent with the findings of the TONIC trial,^[18]^ in which short-term platinum induction enhanced immune-related signatures and was proposed to function as an immunomodulatory primer that could increase tumor sensitivity to immune checkpoint inhibition.

**Table 1:** Summary of the patient’s clinical course and treatment timeline.

Line of therapy	Systemic regimen	Duration (DFI/PFS)	Key radiologic evidence	Neurologic function (MRC scale)
Adjuvant (October 2017–November 2019)	Liposomal paclitaxel + cyclophosphamide (6 cycles)	DFI = 24 M	Chest wall recurrence, occipital bone metastasis (Fig. 1A and B)	Normal
1L (December 2019–September 2020)	Liposomal doxorubicin	PFS = 9 M	New LM surrounding the cauda equina (Fig. 1D)	Symptom onset (complete paralysis)
2L (November 2020–January 2021)	Nab-paclitaxel + tislelizumab	PFS = 2 M	New clival lesion (Fig. 1E), mild LM progression (Fig. 1F)	Brief improvement → 0 (paralysis)
3L (January 2021–August 2021)	Carboplatin + tislelizumab (carboplatin stopped after 2 M due to toxicity) → tislelizumab monotherapy (5 M)	PFS = 7 M	Partial relief of clival/occipital bone lesions (Fig. 1G), new proximal left femoral lesion (Fig. 1H)	0 → 2 (sustained 7 M) → 0 (deterioration)
4L (September 2021–January 2025)	Olaparib + tislelizumab	PFS = 40 M	Marked regression of LM (Fig. 1I), resolution of femoral lesion (Fig. 1J)	0 → 2 (sustained stable)

Bone-modifying therapy was maintained throughout all subsequent lines of treatment for metastatic disease with bone involvement.

1L = first-line, 2L = second-line, 3L = third-line, 4L = fourth-line, DFI = disease-free interval, LM = leptomeningeal metastasis, M = month, MRC = Medical Research Council, PFS = progression-free survival.

The most striking inflection in this patient’s trajectory occurred after integration of pharmacogenomic data into therapeutic planning. At that time, evidence supporting PARP–ICI combinations in *gPALB2*-mutated breast cancer was scarce. The treatment decision was informed by prior signals of efficacy from the TOPACIO/KEYNOTE-162 and MEDIOLA trials in *gBRCA*-mutated disease,^[19,20]^ together with the TBCRC 048 study demonstrating olaparib monotherapy activity in *gPALB2* tumors.^[11]^ These findings suggested that *PALB2*-driven homologous recombination deficiency may confer a “BRCA-like” therapeutic vulnerability,^[21]^ providing a biological rationale for PARP–PD-1 synergy. Continuation of PD-1 blockade in the fourth-line setting was an individualized, evidence-informed decision based on the patient’s prior clinically meaningful neurological improvement, favorable tolerability, and absence of immune-related adverse events. Thus, continuing PD-1 blockade while introducing a pharmacogenomically matched PARP inhibitor represented a genotype-informed, low-toxicity strategy and raised the hypothesis that selected patients may retain potential benefit from immune checkpoint inhibition beyond progression.

Recent findings from 2 phase 2 trials have further informed the clinical context of PARP–PD-1 combinations. KEYLYNK-007 reported antitumor activity with olaparib plus pembrolizumab in patients with HRR-mutated and/or homologous recombination deficiency-positive advanced solid tumors,^[22]^ including *gPALB2*-mutated tumors. KEYLYNK-009 showed that post-induction pembrolizumab plus olaparib achieved PFS comparable to pembrolizumab plus chemotherapy in patients with TNBC,^[23]^ with a numerically favorable trend among those with tumor *BRCA1/2* mutations. Participants in KEYLYNK-007 were immunotherapy-naïve, whereas KEYLYNK-009 introduced olaparib only in patients who had not progressed during first-line platinum-plus-pembrolizumab induction. Furthermore, neither trial specifically evaluated patients with central nervous system (CNS) metastases. However, our patient had spinal leptomeningeal involvement and received olaparib while continuing tislelizumab after overall disease progression during PD-1 blockade maintenance. This clinically distinct, hypothesis-generating observation suggests that PARP inhibitor–based strategies and continuation of PD-1 blockade beyond progression warrant further investigation in carefully selected patients with *gPALB2*-mutated TNBC and CNS involvement.

Preclinical studies suggest that PARP inhibition may enhance tumor immunogenicity and activate interferon signaling, thereby priming the tumor microenvironment for PD-1 blockade.^[24]^ However, these mechanisms were not directly evaluated in this patient. Although the *gPALB2* mutation provided a biological rationale for olaparib treatment, the relative contributions of PARP inhibition, continued PD-1 blockade, and prior platinum exposure to the unusually prolonged PFS of approximately 40 months cannot be determined from a single case. Furthermore, while the patient’s neurological recovery (lower-limb strength improvement from MRC grade 0 to grade 2) represents a clinically meaningful functional outcome, the Response Assessment in Neuro-Oncology criteria for LM recommend an integrated assessment combining neurological, neuroradiographic, and cerebrospinal fluid evaluations.^[25]^ Because the patient declined serial contrast-enhanced neuroimaging and lumbar puncture, formal Response Assessment in Neuro-Oncology criteria for LM response classification could not be performed. Overall, definitive conclusions regarding treatment efficacy are limited by the single-case nature of this report.

## 4. Conclusion

In conclusion, pharmacogenomic profiling identified a potentially actionable *gPALB2* mutation and informed the addition of olaparib to continued tislelizumab in this patient with advanced TNBC and leptomeningeal involvement. This strategy was associated with neurological improvement, acceptable tolerability, and an approximately 40-month PFS after overall disease progression during PD-1 blockade maintenance. As a single-case observation, these findings should be interpreted cautiously and cannot establish the independent contribution of any treatment component. Nevertheless, this hypothesis-generating observation suggests that PARP inhibitor–based treatment and continuation of PD-1 blockade beyond progression warrant further investigation in carefully selected patients with *gPALB2*-mutated TNBC, particularly those with CNS metastasis.

## Acknowledgments

We sincerely thank the patient and her family for their generous support and cooperation. In April 2024, they agreed to undergo enhanced imaging assessments to document objective evidence of treatment efficacy, which greatly enriched the scientific rigor of this report. Their contribution was invaluable to the completion of this work.

## Author contributions

**Conceptualization:** Yulei Wang, Yifang Zhang, Yuxia Chen, Guochun Zhang.

**Funding acquisition:** Yulei Wang, Yifang Zhang, Guochun Zhang.

**Investigation:** Yulei Wang, Jiali Lin, Yi Zhang, Xin Lin, Xiangqing Chen, Lijuan Guo, Yifang Zhang, Yuxia Chen, Guochun Zhang.

**Project administration:** Guochun Zhang.

**Resources:** Bo Liang.

**Supervision:** Guochun Zhang.

**Validation:** Yi Zhang.

**Visualization:** Yulei Wang, Jiali Lin, Yi Zhang, Xin Lin, Xiangqing Chen, Lijuan Guo, Yifang Zhang, Yuxia Chen, Guochun Zhang.

**Writing – original draft:** Yulei Wang, Jiali Lin, Yi Zhang, Bo Liang, Xin Lin, Xiangqing Chen, Lijuan Guo, Yifang Zhang, Yuxia Chen, Guochun Zhang.

**Writing – review & editing:** Yulei Wang, Jiali Lin, Yi Zhang, Bo Liang, Xin Lin, Xiangqing Chen, Lijuan Guo, Yifang Zhang, Yuxia Chen, Guochun Zhang.

## References

[1] Wang X Li X Dong T. Global biomarker trends in triple-negative breast cancer research: a bibliometric analysis. Int J Surg. 2024;110:7962–83. 38857504 10.1097/JS9.0000000000001799 PMC11634138

[2] Raman R Debata S Govindarajan T Kumar P. Targeting triple-negative breast cancer: resistance mechanisms and therapeutic advancements. Cancer Med. 2025;14:e70803. 40318146 10.1002/cam4.70803 PMC12048392

[3] Li Y Zhang H Merkher Y. Recent advances in therapeutic strategies for triple-negative breast cancer. J Hematol Oncol. 2022;15:121. 36038913 10.1186/s13045-022-01341-0 PMC9422136

[4] Liu Y Hu Y Xue J. Advances in immunotherapy for triple-negative breast cancer. Mol Cancer. 2023;22:145. 37660039 10.1186/s12943-023-01850-7 PMC10474743

[5] Tutt ANJ Garber JE Kaufman B. Adjuvant olaparib for patients with BRCA1- or BRCA2-mutated breast cancer. N Engl J Med. 2021;384:2394–405. 34081848 10.1056/NEJMoa2105215 PMC9126186

[6] Robson M Im SA Senkus E. Olaparib for metastatic breast cancer in patients with a germline BRCA mutation. N Engl J Med. 2017;377:523–33. 28578601 10.1056/NEJMoa1706450

[7] Rahman N Seal S Thompson D. PALB2, which encodes a BRCA2-interacting protein, is a breast cancer susceptibility gene. Nat Genet. 2007;39:165–7. 17200668 10.1038/ng1959 PMC2871593

[8] Foo TK Xia B. BRCA1-dependent and independent recruitment of PALB2-BRCA2-RAD51 in the DNA damage response and cancer. Cancer Res. 2022;82:3191–7. 35819255 10.1158/0008-5472.CAN-22-1535 PMC9481714

[9] Cybulski C Kluzniak W Huzarski T. Clinical outcomes in women with breast cancer and a PALB2 mutation: a prospective cohort analysis. Lancet Oncol. 2015;16:638–44. 25959805 10.1016/S1470-2045(15)70142-7

[10] Turk AA Wisinski KB. PARP inhibitors in breast cancer: bringing synthetic lethality to the bedside. Cancer. 2018;124:2498–506. 29660759 10.1002/cncr.31307 PMC5990439

[11] Tung NM Robson ME Ventz S. TBCRC 048: phase II study of olaparib for metastatic breast cancer and mutations in homologous recombination-related genes. J Clin Oncol. 2020;38:4274–82. 33119476 10.1200/JCO.20.02151

[12] Kuemmel S Harrach H Schmutzler RK. Olaparib for metastatic breast cancer in a patient with a germline PALB2 variant. NPJ Breast Cancer. 2020;6:31. 32728620 10.1038/s41523-020-00174-9 PMC7382468

[13] Zhang G Ren C Li C. Distinct clinical and somatic mutational features of breast tumors with high-, low-, or non-expressing human epidermal growth factor receptor 2 status. BMC Med. 2022;20:142. 35484593 10.1186/s12916-022-02346-9 PMC9052533

[14] Chen B Zhang G Li X. Comparison of BRCA versus non-BRCA germline mutations and associated somatic mutation profiles in patients with unselected breast cancer. Aging (Albany NY). 2020;12:3140–55. 32091409 10.18632/aging.102783 PMC7066887

[15] Emens LA Adams S Barrios CH. First-line atezolizumab plus nab-paclitaxel for unresectable, locally advanced, or metastatic triple-negative breast cancer: IMpassion130 final overall survival analysis. Ann Oncol. 2021;32:983–93. 34272041 10.1016/j.annonc.2021.05.355

[16] Cortes J Cescon DW Rugo HS. Pembrolizumab plus chemotherapy versus placebo plus chemotherapy for previously untreated locally recurrent inoperable or metastatic triple-negative breast cancer (KEYNOTE-355): a randomised, placebo-controlled, double-blind, phase 3 clinical trial. Lancet. 2020;396:1817–28. 33278935 10.1016/S0140-6736(20)32531-9

[17] Yu Z. Tislelizumab: an effective treatment option for early-stage triple-negative breast cancer. Transl Breast Cancer Res. 2024;5:5. 38751685 10.21037/tbcr-23-39 PMC11093077

[18] Voorwerk L Slagter M Horlings HM. Immune induction strategies in metastatic triple-negative breast cancer to enhance the sensitivity to PD-1 blockade: the TONIC trial. Nat Med. 2019;25:920–8. 31086347 10.1038/s41591-019-0432-4

[19] Vinayak S Tolaney SM Schwartzberg L. Open-label clinical trial of niraparib combined with pembrolizumab for treatment of advanced or metastatic triple-negative breast cancer. JAMA Oncol. 2019;5:1132–40. 31194225 10.1001/jamaoncol.2019.1029 PMC6567845

[20] Domchek SM Postel-Vinay S Im SA. Olaparib and durvalumab in patients with germline BRCA-mutated metastatic breast cancer (MEDIOLA): an open-label, multicentre, phase 1/2, basket study. Lancet Oncol. 2020;21:1155–64. 32771088 10.1016/S1470-2045(20)30324-7

[21] Guo M Wang SM. The BRCAness landscape of cancer. Cells. 2022;11:3877. 36497135 10.3390/cells11233877 PMC9738094

[22] Yap TA Shapira-Frommer R Lugowska I. Abstract CT004: KEYLYNK-007: tumor agnostic trial of olaparib plus pembrolizumab in homologous recombination repair mutation- and homologous recombination deficiency-positive advanced cancers. Cancer Res. 2025;85:CT004.

[23] Rugo HS Cescon DW Robson ME. KEYLYNK-009: pembrolizumab plus olaparib in locally recurrent inoperable or metastatic triple-negative breast cancer after clinical benefit from first-line pembrolizumab plus chemotherapy. Clin Cancer Res. 2026;32:883–93. 41405563 10.1158/1078-0432.CCR-25-1818

[24] Zhou T Zhang J. Therapeutic advances and application of PARP inhibitors in breast cancer. Transl Oncol. 2025;57:102410. 40359851 10.1016/j.tranon.2025.102410 PMC12142329

[25] Chamberlain M Junck L Brandsma D. Leptomeningeal metastases: a RANO proposal for response criteria. Neuro Oncol. 2017;19:484–92. 28039364 10.1093/neuonc/now183 PMC5464328

